# Metachronous early gastric cancer successfully treated twice with endoscopic submucosal dissection: A case report

**DOI:** 10.1097/MD.0000000000045017

**Published:** 2025-10-10

**Authors:** Xiaoyun Wang, Ru Ma, Lu Yuan, Jingjing Li, Guolong Li, Xiaojin Ma, Na Tang

**Affiliations:** a Department of Gastroenterology, The First People’s Hospital of Shizuishan, Affiliated to Ningxia Medical University, Shizuishan, China.

**Keywords:** case report, early gastric cancer, endoscopic submucosal dissection, metachronous gastric cancer

## Abstract

**Rationale:**

Metachronous early gastric cancer may arise years after curative endoscopic resection of an index lesion. Evidence on the feasibility of a second endoscopic submucosal dissection (ESD) remains limited.

**Patient concerns:**

A 45-year-old asymptomatic man underwent routine surveillance 5 years after curative ESD for a well-differentiated intramucosal tubular adenocarcinoma of the mid-gastric body.

**Diagnoses:**

Magnifying narrow band imaging revealed a 20-mm flat erythematous lesion on the anterior wall of the gastric angle. Biopsy confirmed well-differentiated tubular adenocarcinoma confined to the mucosa.

**Interventions:**

Repeat ESD achieved en-bloc resection with negative horizontal and vertical margins. Histopathology showed intramucosal carcinoma without lymphovascular invasion (eCuraA).

**Outcomes:**

The patient recovered uneventfully and remained disease-free at 12-month follow-up.

**Lessons:**

For patients with metachronous early gastric cancer meeting curative endoscopic resection criteria, a second ESD is technically feasible, oncologically curative, and preferable to surgery, sparing them the significant morbidity of gastrectomy.

## 1. Introduction

Early gastric cancer (EGC) is histologically defined as adenocarcinoma confined to the mucosal or submucosal layers, regardless of lymph node status. When resected with tumor-free margins, this stage carries an excellent prognosis: contemporary Asian and Western cohorts report 5-year disease-specific survival rates consistently >90%.^[1]^ Such outcomes highlight the biologic indolence of intramucosal lesions and the efficacy of modern endoscopic management.

Endoscopic submucosal dissection (ESD) has become the organ-preserving gold standard for EGC that fulfills absolute or carefully vetted expanded criteria. Unlike conventional endoscopic mucosal resection, ESD allows controlled circumferential incision and submucosal dissection, yielding an en-bloc specimen irrespective of lesion size, location, or fibrosis. This approach delivers R0 resection rates exceeding 95% in experienced centers, permits meticulous histopathological assessment of invasion depth, differentiation, and lymphovascular infiltration, and obviates the morbidity of gastrectomy for most patients.^[2]^ Procedure-related bleeding or perforation occurs in ≤5%, and is usually managed endoscopically, preserving the minimally invasive nature of the technique. However, curative ESD does not eliminate oncogenic field changes within the gastric mucosa—chiefly *Helicobacter pylori*-driven chronic atrophic gastritis and intestinal metaplasia. Consequently, metachronous EGC, defined as a new carcinoma arising ≥12 months after index resection, develops in 2.8% to 15.9% of patients within 10 years.^[3]^

We present a patient who underwent 2 curative ESDs 5 years apart, highlighting key clinical decision-making principles, endoscopic techniques, pathological findings, and long-term surveillance strategies for optimal management of metachronous lesions.

## 2. Case presentation

**Figure 1.**
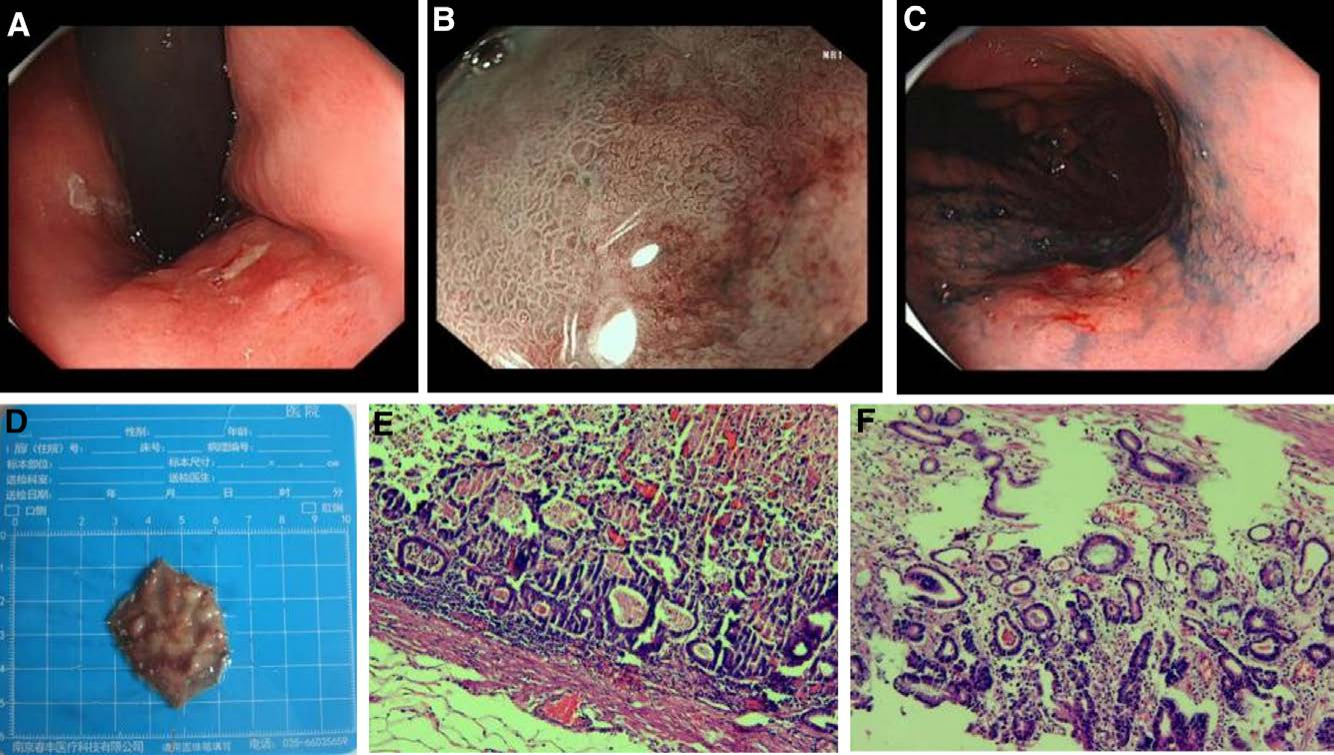
Index early gastric cancer (2018). (A) White-light EGD showing an 8 mm reddish depressed lesion on the mid-gastric body. (B) Magnifying narrow band imaging revealed an irregular microvascular pattern with clear demarcation line. (C) Indigo carmine chromoendoscopy enhanced lesion borders. (D) En-bloc specimen after ESD (3 × 4 cm). (E) Histology (H&E, ×100) showing well-differentiated tubular adenocarcinoma confined to the mucosa. (F) Higher magnification (×200) illustrating irregular fused glands. EGD = oesophagogastroduodenoscopy, ESD = endoscopic submucosal dissection.

A 45-year-old man with well-controlled hypertension and type 2 diabetes mellitus but no *Helicobacter pylori* infection or family history of gastric cancer underwent screening oesophagogastroduodenoscopy (EGD) in September 2018. High-definition white-light imaging identified an 8 × 6 mm depressed (0-IIc) reddish lesion on the mid-gastric body (Fig. 1A). Chromoendoscopy with indigo carmine and magnifying narrow band imaging delineated clear margins and an irregular microsurface/microvascular pattern (Fig. 1B, C). Endoscopic ultrasound was not required because pit-plus-vascular patterns suggested intramucosal invasion. En-bloc ESD (Fig. 1D) was performed using a dual knife following submucosal injection of glycerol plus epinephrine–indigo carmine solution. Haemostasis was achieved by hot biopsy forceps. Histology disclosed a well-differentiated tubular adenocarcinoma limited to the mucosa (pT1a) with negative lateral and vertical margins and no lymphovascular invasion (eCuraA) (Fig. 1E, F). The patient commenced annual EGD surveillance as per institutional protocol.

**Figure 2.**
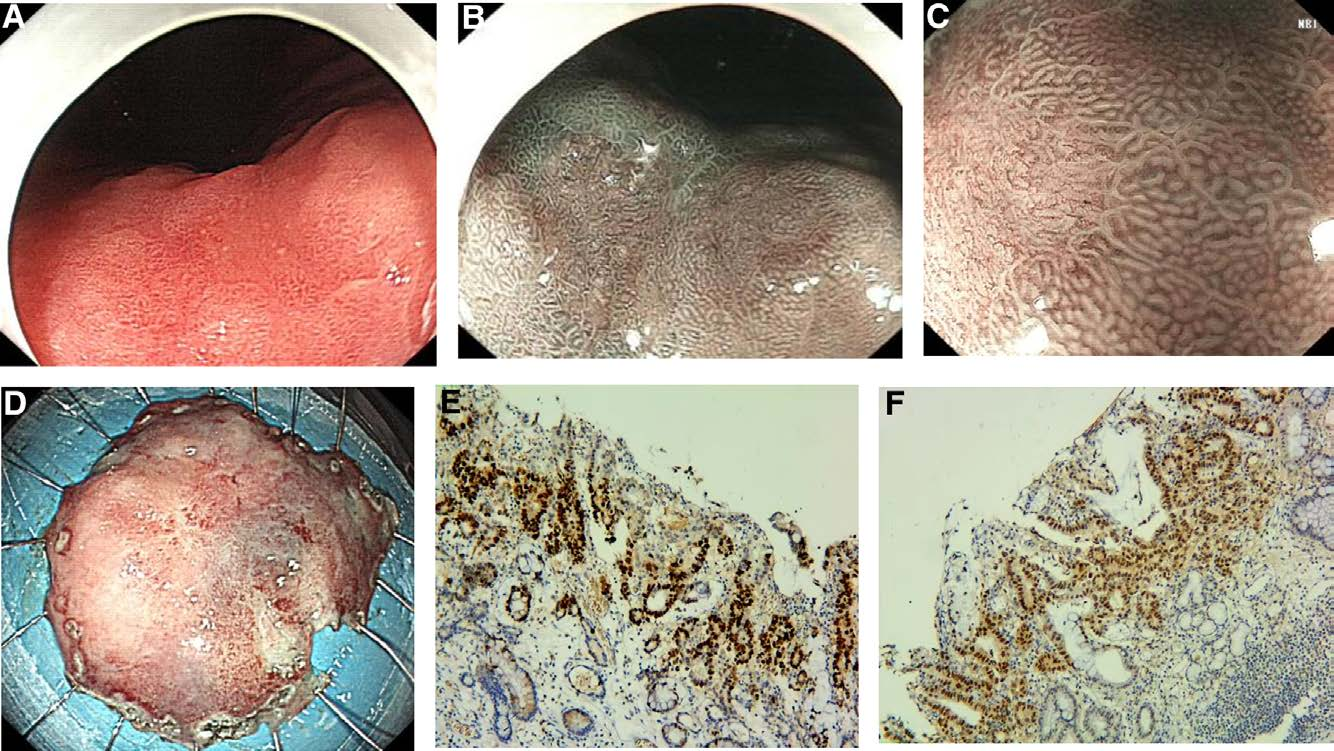
Metachronous lesion and second ESD (2023). (A) White-light EGD depicting a 20 mm flat erythematous lesion on the anterior gastric angle. (B) NBI weak magnification showing mesh-like microvessels. (C) NBI high magnification confirming irregular microsurface and microvascular pattern with clear boundary. (D) Resected specimen (3 × 4 cm) after second ESD. (E) Histology (H&E, ×100) revealing well-differentiated intramucosal adenocarcinoma. (F) Histology (×200) displaying glands of varying sizes with disorganized architecture and areas of confluence. EGD = oesophagogastroduodenoscopy, ESD = endoscopic submucosal dissection, NBI = narrow band imaging.

In September 2023, routine surveillance EGD identified a 20 × 16 mm flat erythematous lesion on the anterior wall of the gastric angle (Fig. 2A–C). Biopsies revealed well-differentiated adenocarcinoma. Contrast-enhanced abdominal computed tomography excluded nodal or distant metastases. Following multidisciplinary team discussion and comprehensive evaluation against current guidelines, repeat ESD under general anesthesia achieved R0 resection (Fig. 2D). Pathology confirmed a well-differentiated tubular adenocarcinoma confined to the mucosa (0-IIa), margins negative, no lymphovascular invasion, chronic atrophic gastritis with intestinal metaplasia in adjacent mucosa (Fig. 2E, F). The second procedure lasted 65 minutes with minimal blood loss. Proton-pump inhibitors were prescribed for 8 weeks.

At 3, 6, and 12 months post-second ESD, EGD showed mature scar tissue without recurrence; abdominal computed tomography was unremarkable. The patient remains asymptomatic and under annual surveillance.

## 3. Discussion

Early gastric cancer represents invasive adenocarcinoma confined to the mucosa and submucosa, classified as T1N0-3M0 regardless of lymph node metastasis.^[4,5]^ The subtle endoscopic appearance of EGC under conventional white-light imaging often mimics benign inflammatory changes, necessitating image-enhanced endoscopy for accurate detection chromoendoscopy using contrast agents such as indigo carmine enhances mucosal surface details and lesion demarcation, facilitating precise determination of resection margins. Electronic chromoendoscopy, particularly narrow band imaging, provides real-time visualization of microsurface and microvascular patterns without requiring dye application, enabling efficient differentiation between neoplastic and nonneoplastic tissue.^[2]^

The choice between repeat endoscopic resection and surgical gastrectomy for metachronous EGC requires careful consideration of multiple factors based on current evidence and established guidelines. The Japanese Gastric Cancer Association and the European Society of Gastrointestinal Endoscopy clearly define absolute indications for ESD: well-differentiated intramucosal adenocarcinoma ≤2 cm without ulceration, or differentiated intramucosal carcinoma >2 cm without ulceration and lymphovascular invasion.^[2,4]^ Our patient’s metachronous lesion fulfilled these absolute criteria, making repeat ESD the preferred therapeutic approach. Several factors supported this clinical decision: the lesion met absolute indications with favorable histological characteristics; the patient’s young age and excellent performance status; the technical feasibility demonstrated by successful prior ESD; the patient’s preference to avoid surgical morbidity; and the absence of contraindications to endoscopic therapy. Recent studies have demonstrated that repeat ESD achieves comparable oncological outcomes to initial procedures, with en-bloc resection rates of 95% to 98% and negligible procedure-related mortality.^[6,7]^ In contrast, distal gastrectomy, while curative, carries operative mortality of 0.5% to 1% and significant long-term morbidity including dumping syndrome, nutritional deficiencies, bone disease, and substantially reduced quality of life. The organ-preserving advantage of ESD is particularly valuable in young patients with long life expectancy, as demonstrated in our case.

Endoscopic resection of appropriately selected EGC achieves equivalent oncological outcomes to surgical resection, while preserving organ function and minimizing morbidity.^[1,5]^ However, strict adherence to established guidelines remains paramount, requiring comprehensive preoperative assessments of histological differentiation, lesion size, ulceration status, and invasion depth.^[2,4,8]^ The present case exemplifies successful application of these evidence-based selection criteria.

The post-resection curability assessment utilizes the established eCuraA classification system, which stratifies patients based on histological risk factors and guides subsequent management decisions. eCuraA status, achieved in both procedures in our patient, indicates curative resection with minimal risk of residual disease, requiring only endoscopic surveillance without additional therapy. The reported incidence of metachronous gastric neoplasia ranges from 2.8% to 15.9%, with cumulative rates of 9.5%, 13.1%, and 22.7% at 5, 7, and 10 years, respectively.^[6–8]^ These data underscore the critical importance of long-term surveillance protocols in detecting second primary tumors at a curable stage. This case demonstrates the technical feasibility, safety, and oncological efficacy of repeat ESD for metachronous EGC when appropriate selection criteria are rigorously applied. The continuous evolution of endoscopic techniques and instrumentation expands the applicability of organ-preserving therapy, offering patients curative treatment while maintaining optimal quality of life.

## 4. Conclusion

Stringent long-term endoscopic surveillance facilitated early detection of metachronous early gastric cancer 5 years after initial curative ESD. Repeat ESD proved technically safe and oncologically curative, reinforcing the central role of organ-preserving endoscopic therapy in the management of appropriately selected metachronous lesions.

## Acknowledgments

We thank for Dr Bin Qiao for editing this manuscript.

## Author contributions

**Formal analysis:** Ru Ma, Lu Yuan, Jingjing Li, Guolong Li, Xiaojin Ma, Na Tang.

**Writing – original draft:** Xiaoyun Wang, Ru Ma, Lu Yuan.

**Writing – review & editing:** Xiaoyun Wang.
